# Alleviation of Antioxidant Defense System by Ozonized Olive Oil in DNBS-Induced Colitis in Rats

**DOI:** 10.1155/2014/967205

**Published:** 2014-09-04

**Authors:** Eman Abu-Gharbieh, Fatehia A. Bayoumi, Naglaa G. Ahmed

**Affiliations:** ^1^Department of Pharmacology and Toxicology, Dubai Pharmacy College, P.O. Box 19099, Dubai, UAE; ^2^Department of Pathology, Dubai Medical College, Dubai, UAE; ^3^Department of Pharmaceutical Chemistry and Natural Products, Dubai Pharmacy College, Dubai, UAE

## Abstract

The aim of the study was to evaluate the potential protective effect of ozonized olive oil (OZO) in 2,4-dinitrobenzene sulphuric acid (DNBS) induced colitis in rats and to elucidate the role of some antioxidant defense system (superoxide dismutase “SOD,” glutathione peroxidase “GSH-Px,” and catalase “CAT”) in these effects. The physicochemical parameters including viscosity, peroxide, and acid values of olive oil and OZO were evaluated. The animals were divided into several groups and the colitis was induced in the rats by intracolonic instillation of DNBS at dose of 15 mg/rat. Olive oil (OO) at dose of 6 mg/kg and OZO at doses of 3 and 6 mg/kg was administered orally for 7 days, starting the day before induction of colitis. Our results showed that macroscopic and microscopic damage scores were significantly reduced in a dose response manner in rats pretreated with OZO only. In contrast, CAT, GSH-Px, and SOD activities were significantly increased in the distal colon of inflamed animals pretreated with OZO with respect to control group dose dependently. Results demonstrate that OZO pretreatment exerts protective effects in DNBS induced colitis in rats and provide evidence that the protective effects of OZO are mediated by stimulation of some antioxidant enzymes.

## 1. Introduction

Ozonized Olive Oil (OZO) is a powerful natural remedy for a variety of health concerns, especially those related to skin health [1]. OZO is a registered drug and has been used effectively for healing wounds and pressure ulcers in mice [2]. Clinically, OZO has been used to treat bedsores, intractable fistula and wounds after surgical operation [3].

The olive oil (OO) is obtained from the fruit of the olive tree. OO contains different fatty acids. Oleic acid (65–85%) was detected as the main fatty acid along with 10% linoleic acid, 9% palmitic acid, and 3% stearic acid [4].

OZO is produced by the reaction of ozone with the olive oil almost exclusively with the carbon-carbon double bonds present in the unsaturated fatty acids. This reaction produces several oxygenated compounds such as hydroperoxides, ozonides, aldehydes, peroxides, diperoxides, and polyperoxides [5]. Triolein triozonide (ozonized triolein) has been identified as the major oxygenated triglyceride in OZO [6]. OZO may be slowly decomposed due to the inherent structure of the triolein triozonide molecule. This decomposition can be delayed by avoiding moisture and storing the oil at low temperature. The oxygenated compounds could be responsible for the wide biological activity of OZO. In a few clinical cases in Japan, OZO has been used to treat bedsores and intractable fistula or wounds after surgical operation [3, 8]; however, the detailed action mechanism of OZO is not clear. Also, OZO can control the key events in the healing phases and triggering and modulating the inflammatory stage. The aim of this work was to evaluate the potential protective effect of OZO in 2,4-dinitrobenzene sulphuric acid (DNBS) induced colitis in rats as well as elucidate the role of some important constituents of antioxidant defense system such as superoxide dismutase (SOD), glutathione peroxidase (GSH-Px), and catalase (CAT) in these possible effects. Thiobarbituric acid reactive substances (TBARS) were also measured.

## 2. Materials and Methods

### 2.1. Animals

Male albino rats of 200 ± 20 g body weight were used in this study. Animals were housed in a well-controlled environment and had free access to food and water throughout the study period. One week before starting the experimental procedure, animals were weighed and gently manipulated in the laboratory environment for 20 min every day to minimize the effects of stress* per se* on the parameters to be measured.

Animal welfare and experimental procedures were carried out in accordance with the guide for the care and use of laboratory animals [9]. The protocol for induction of colitis was reviewed and approved by the Research Committee of Dubai Pharmacy College.

### 2.2. Solvents and Reagents

All reagents used for the determinations of SOD, CAT, GSHPx, and TBARS were purchased from Sigma Chemicals (St. Louis, Mo, USA). Potassium iodide, sodium thiosulfate, starch, ethanol, potassium hydroxide, and phenolphthalein were purchased from Merck (Germany) and 2,4-dinitrobenzene sulphonic acid (DNBS) from ICN Biomedicals.

Olive oil was obtained from a registered trademark from Palestine. Ozonized oil was kindly donated from Ozone Clinic, Rashid Hospital, Dubai, UAE.

### 2.3. Preparation of Ozonized Oil

Ozonation of olive oil was conducted by the procedure described by Sakazaki et al. [2]. The OZO is formed from pure virgin olive oil and it was subjected to high concentrations of ozone gas for a limited period till it would be transformed to OZO. The OZO was stored at 8–10°C and allowed to melt at room temperature before use.

### 2.4. Analyses of Physicochemical Parameters

They are as follows: (i) Peroxide index (PI) indicates the quantity of peroxide available in OZO [10]; (ii) acid value is the number of milligrams of potassium hydroxide required to neutralize the free acids in 1.0 g of the substance [10]; (iii) viscosity was measured by DV-11 + proviscometer at room temperature.

### 2.5. Experimental Design

Animals were randomly assigned to seven groups with 6 animals each. The noninflamed groups were as follows: nontreated (negative control), OO- and OZO-treated groups at doses of 6 mg/kg each, while the inflamed groups were the nontreated, OO-treated at dose of 6 mg/kg, and OZO-treated at doses of 3 and 6 mg/kg.

Starting the day before colitis induction, the oils treatments were given orally, by gavage, once daily at the same time. The animals' body weights were recorded daily during the experimental period. At day 6 after colitis, the animals were sacrificed by ether overdose.

### 2.6. Induction of Experimental Colitis

Colitis was induced using a previously described method by Vasina et al. [11]. The animals were deprived of food for 24 hours before the colitis induction but had free access to water and the rats were lightly anaesthetized by inhalation of ethyl ether. 2,4-dinitrobenzene sulphonic acid (15 mg per rat) dissolved in 0.25 mL of 50% ethanol was instilled into the distal colon of each animal using a rubber catheter, so that the tip was about 8 cm proximal to the anus. Control rats received 0.25 mL 0.9% NaCl alone intrarectally. 2,4-Dinitrobenzene sulphonic acid and control rats were kept in separated cages during the study.

### 2.7. Tissue Collection

Rats were killed at the sixth day after colitis induction and the distal colon was removed, opened longitudinally, and washed with phosphate-buffered saline (PBS). Whole-wall samples from distal-colon, taken from an area immediately adjacent to the gross macroscopic damage, were cut out and fixed in 10% formalin solution. Sections of 5 *μ*m thickness of colon were cut, serially mounted on glasses, and processed for routine haematoxylin-eosin (H&E) staining. Specimens of colonic tissue were also removed from the area of gross injury, snap frozen in liquid nitrogen, and stored at −80°C until subsequent assays.

### 2.8. Assessment of Colonic Damage

Colonic damage was assessed macroscopically and histologically using a method previously described [11].

The macroscopic features were based on the following features: presence of adhesions between the colon and other intra-abdominal organs, consistency of colonic faecal material, thickening of the colonic wall, presence and extension of hyperaemia. Microscopic criteria for damage and inflammation were assessed by light microscope on H&E stained histological sections. Histological parameters included the degree of mucosal architecture changes, cellular infiltration, external muscle thickening, presence of crypt abscess, and goblet cell depletion.

### 2.9. Biochemical Assays

Colonic tissues were weighted and homogenized in 10% w/v of a solution of KCl 100 mM with EDTA 0.3 mM for both GSH-Px and SOD, while, for the assessment of both CAT and TBARS, tissues were homogenized in 50 mM phosphate buffer (pH 7) using a tissue homogenizer Ultra-turrax T25 Polytron at 4°C then centrifuged at 6000 g at 4°C.

100 *μ*L supernatant was then added to the enzyme immunoassay 96-well plate in duplicate and assayed according to the manufacturers' protocols for GSH-Px, SOD, and CAT assay kits.

Thiobarbituric acid reactive substances (TBARS) levels are considered as lipid peroxides index (LP) as per Ohkawa et al. [12]. Ten percent (w/v) tissue homogenate was mixed with sodium dodecyl sulfate, acetate buffer (pH 3.5), and aqueous solution of thiobarbituric acid. After heating at 95°C for 60 min, the red pigment produced was extracted with* n*-butanol-pyridine mixture and estimated by the absorbance at 532 nm and the results were expressed as nmol of TBA per gram of proteins.

Protein concentrations were determined by the method of Lowry et al. [13] using bovine serum albumin as standard.

### 2.10. Statistical Analysis

Results are expressed as mean values ± standard error mean (SEM). Statistical analysis was performed using One-Way or Two-Way ANOVA, as appropriate, with Bonferroni's correction for multiple comparisons. A *P* value < 0.05 was considered significant. Calculations were performed using GraphPad Prism (version 5.0; GraphPad Software Inc., San Diego, CA, USA).

## 3. Results

The physicochemical parameters of OO and OZO are shown in Table 1.

The exposure of OO to the ozone turned it to yellowish-white semisolid. Moreover, the acid and peroxide values of the ozonized oil were increased.

**Figure 1:**
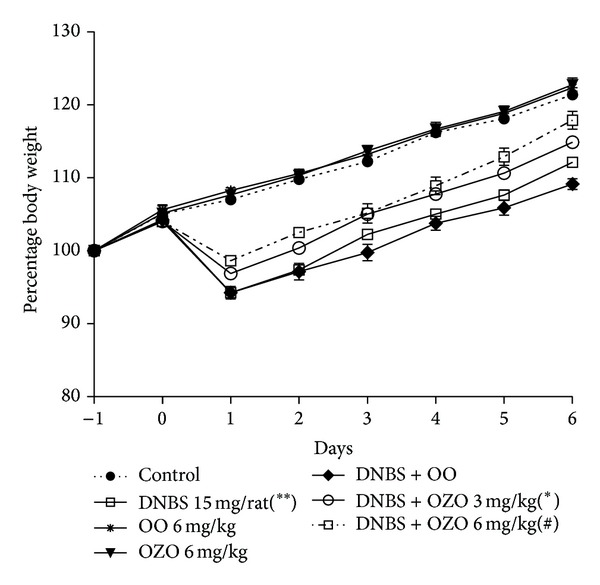
Body weight change in the different experimental groups. Data are expressed as mean values ± SEM. *n* = 6 rats per group. Statistical analysis was performed with Two-Way ANOVA; ∗ and # *P* values are less than 0.05 and 0.01, respectively versus inflamed group; **P < 0.01 versus control group.

Figure 1 is representing the gain in body weight for the animals during the experimental period. Intrarectal administration of DNBS significantly reduced the body weight gain with respect to noninflamed controls (*P* < 0.01). Treatment with OZO at the doses of 3 and 6 mg/kg significantly reduced the impairment in body weight gain induced by colitis dose dependently as shown in (Figure 1), whereas OO has no significant effect on body weight gain compared to its respected controls.

Table 2 summarizes the effects of both OO and OZO on the colonic macro- and microscopic damage scores for the entire groups. The macro- and microscopic damage scores of the inflamed nontreated rats increased by six- and tenfold, respectively, over the negative control animals. Whereas there were no significant effects on both macro- and microscopic damage scores for the noninflamed animals treated with OO and OZO verses the negative control group.

Pretreatment with OO did not show any change in both macro- and microscopic damage score in the inflamed animals (*P* > 0.05). On the other hand, OZO pretreatment at both doses caused significant reduction of both macro- and microscopic damage scores in a dose dependant manner in the inflamed groups as shown in Table 2.

**Figure 2:**
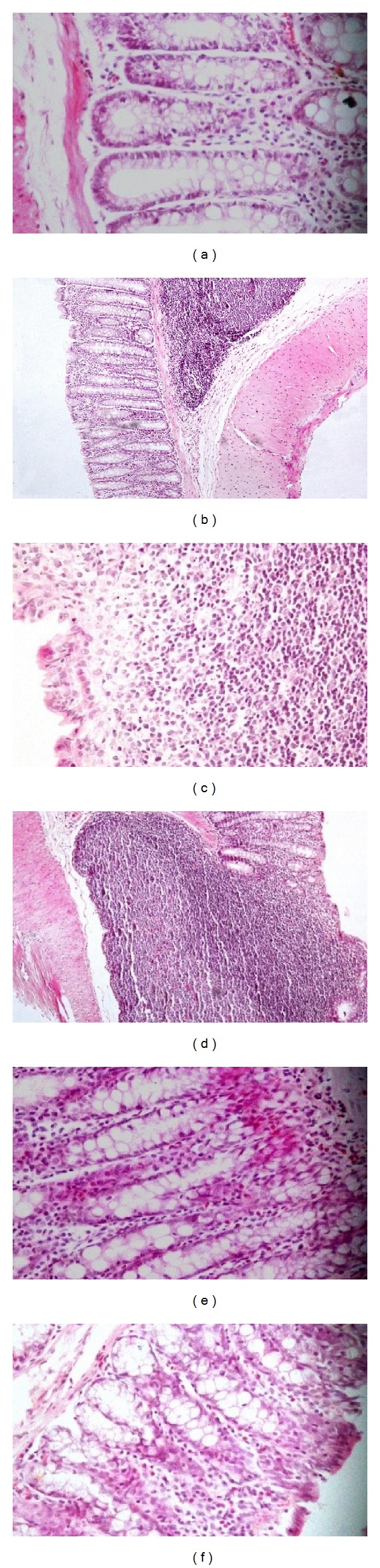
Histopathological findings of colonic tissues from different animals' groups; (a) colonic tissue for animals treated with OO shows normal surface epithelium and crypt cells ×400. (b) Colonic tissue for animals treated with high dose OZO alone with normal surface epithelium and crypt cells ×200. (c) Inflamed control group; the mucosa shows focal loss of the covering and lamina propria shows dense lymphoplasmacytic and histiocytic infiltrate to the submucosa ×200. (d) Inflamed animals treated with OO; mononuclear granulomatous infiltrate encroaching on the overlying mucosa with apparent destruction of the muscularis mucosa and mucosal covering ×200. (e) Inflamed animals treated with low dose OZO nearly normal mucosa and submucosa with polypoid formation. Crypt cell hyperplasia and elongation. Lamina propria is infiltrated by monocytes and eosinophils 400x. (f) Inflamed animals treated with high dose OZO, showing normal mucosal lining and submucosa and cellular infiltrate with eosinophils 200x.

The histological study supports the findings of the damage scores; it shows that OO and OZO administration to the noninflamed animals do not have any destructive effects on the intestinal mucosa (Figures 2(a) and 2(b)). Furthermore, DNBS at dose of 15 mg/rat caused extensive mucosal destruction with well-developed granuloma extending throughout the mucosa and submucosa, often involving the muscularis propria, which invariably appeared thickened (Figure 2(c)). Pretreating the inflamed animals with the OO did not show any improvement in the histological appearance over the inflamed nontreated group (Figure 2(d)). Nevertheless, OZO treatment at both doses showed significant improvement in the histological appearance of the mucosa and submucosa lining to be nearly normal as shown in Figures 2(e) and 2(f). The results of histological study confirmed clearly the protective effect of OZO against colitis induced by DNBS.

Table 3 also shows that DNBS induced a remarkable and significant decrease of GSH-Px, CAT, and SOD activities in rats' colonic tissues, *P* < 0.05.

The lipid peroxides index represented by TBARS content in the colonic tissue increased significantly (*P* < 0.05) from basal concentration of 0.056 ± 0.10 nmol/g of protein to 0.67 ± 0.30 nmol/g of protein in the inflamed nontreated animals. On the other hand, pretreating the inflamed animals with OO did not raise the levels of the three antioxidant enzymes and similarly did not reduce the levels of TBARS in their colonic tissues in respect to the inflamed control (Table 3). On the contrary, administration of OZO to the inflamed rats at both doses increased significantly the SOD and GSH-Px levels dose dependently, while only OZO at the highest dose was significantly able to increase the level of CAT (*P* < 0.05) as shown in Table 3.

Administration of OO* per se* to noninflamed rats had no significant effects on all of the three enzymes and TBARS. Conversely, administration of ozonized oil caused slight increase in the TBARS content in the tissue of noninflamed animals, but this increment was not statistically significant.

## 4. Discussion

The pathogeneses of inflammatory bowel diseases (IBD), including Crohn's disease and ulcerative colitis, are associated with elevated levels of reactive oxygen species (ROS) such as peroxide anion, hydrogen peroxide (H_2_O_2_), and hypochlorous acid [14–16], and this could be attributed to the massive infiltration of polymorphonuclear and mononuclear leukocytes. In fact, free radical production is a key mechanism for the development of colonic inflammation in experimental colitis models as well [17–19]. Hawkins et al. [20] reported that the injury caused by DNBS closely resembles human ulcerative colitis. This model resembles histological features of the human ulcerative colitis in many features like transmural inflammation with granuloma and diffuse ulceration and inflammation and plasma-lymphoid and eosinophil infiltrates, as well as crypt distortion [21].

To regulate overall ROS levels, the intestinal mucosa possesses a complex of antioxidant defense system, of which the SOD, CAT, and GSH-Px are the major players, as SOD is an important radical superoxide scavenger while CAT and GSH-Px are involved in the elimination of hydrogen peroxide and lipid hydroperoxides [22, 23].

The efficacy of the antioxidant system is impaired during inflammation status, partially as a result of autooxidation. In a previous study, Loguercio et al. [24] showed increased level of lipid peroxidation in colonic mucosa treated with TNBS.

On the other hand, ozonized oil is reported to be effective for wounds' healing [7] and pressure ulcers in mice [2] and ethanol-induced ulcers in rats [25]. In addition to ozonides, during the procedure of oil ozonization, many oxygenated compounds are generated like peroxides and aldehydes [26]. These oxygenated compounds may be responsible for many biological activities of ozonized vegetable oils, such as antimicrobial [27] and antifungal activities [28].

Our findings showed that DNBS increases lipid peroxidations with respect to the nontreated group, but no significant changes were found with respect to OZO-inflamed treated animals. This could be explained based on the fact of presence of many oxygenated compounds other than ozonides that have been generated during the ozonization process of the oil such as hydroperoxides and aldehydes; this might contribute to the increased TBARS content in rat colonic mucosa.

The results also revealed a decrease of SOD, CAT, and GSH-Px activities in colonic mucosa of the inflamed control animals, while the levels of both SOD and GSH-Px were increased significantly in rats treated with OZO in a dose dependent manner. CAT activity was significantly increased by the treatment with the highest dose of OZO only, and this finding appears to be due to the fact that CAT has a lower affinity for that ROS comparing to SOD and GSH-Px. This result comes in concordances with many previous studies that reported that GSH-Px plays a much greater role in the removal of H_2_O_2_ than CAT [25, 29, 30].

## 5. Conclusion

The results of this study suggest that the OZO has a protective effect in the rat colonic mucosal damage induced by DNBS and this might be mediated at least partially by its enhancement effect on antioxidant enzymes such as SOD and GSH-Px which comprise the endogenous scavengers of ROS.

## Figures and Tables

**Table 1:** The physicochemical parameters of olive oil and ozonized olive oil.

Item	Olive oil	Ozonized olive oil
Colour	Dark green	Yellowish white
Viscosity cP	8500	semisolid
Peroxide value	1.33	400
Acid value	1%	14.6%

**Table 2:** Macro and microscopic damage scores for the animal groups.

Treatment	Noninflamed (intrarectal vehicle)			Inflamed (intrarectal DNBS 15 mg per rat)			
	Nontreated	OO (6 mg/kg)	OZO (6 mg/kg)	Nontreated	OO (6 mg/kg)	OZO (3 mg/kg)	OZO (6 mg/kg)
Macroscopic score	1.20 ± 0.12	1.11 ± 0.22	1.13 ± 0.16	7.5 ± 0.41^a^	7.4 ± 0.35	5.56 ± 0.15^b^	3.52 ± 0.22^b^
Microscopic score	0.42 ± 0.13	0.38 ± 0.25	0.41 ± 0.28	4.52 ± 0.21^a^	4.31 ± 0.42	3.20 ± 0.27^b^	3.00 ± 0.24^c^

^a^ *P* < 0.001 versus intrarectal vehicle and ^b and c^ *P* < 0.05 and 0.01 versus inflamed non-treated group respectively.

Macroscopic assessment criteria: presence of adhesions (0–2), faecal material consistency (0–2), bowel thickness (mm), presence and extension of hyperaemia (0–6).

Microscopic assessment criteria: degree of mucosal architecture loss (0–3), cellular infiltration (0–3), muscle thickening (0–3), presence of crypt abscess and goblet cell depletion (0-1).

**Table 3:** Effects of OO and OZO in noninflamed and inflamed rats.

Treatment	Noninflamed (intrarectal vehicle)			Inflamed (intrarectal DNBS 15 mg per rat)			
	Nontreated	OO (6 mg/kg)	OZO (6 mg/kg)	Nontreated	OO (6 mg/kg)	OZO (3 mg/kg)	OZO (6 mg/kg)
GSH-Px U/g prot.	1.29 ± 0.14	2.09 ± 0.37	2.9 ± 0.26	0.75 ± 0.11^d^	0.76 ± 0.14	0.84 ± 0.13^a^	1.22 ± 0.15^b^
CAT (KU/gr prot.)	493.48 ± 11.87	523.23 ± 18.04	541 ± 19.65	366.40 ± 17.52^d^	384.85 ± 11.16	431.88 ± 22.72	487.12 ± 17.79^c^
SOD (U/g)	62.50 ± 5.44	72.41 ± 9.35	74.52 ± 6.24	49.33 ± 3.16^d^	56.75 ± 2.35	69.01 ± 0.19^a^	78.67 ± 3.71^b^
TBARS (nmol/g prot.)	0.056 ± 0.10	0.042 ± 0.26	0.12 ± 0.09	0.67 ± 0.30^d^	0.632 ± 0.26	0.619 ± 0.32	0.624 ± 0.18

^a, b, and c^ *P* < 0.01, 0.001, 0.05, respectively, versus inflamed nontreated group.

^d^ *P* < 0.05 versus intrarectal vehicle.

## References

[1] Abeck D, Plötz S (2008). Colloidal silver and ozonized olive oil for atopic dermatitis?. *Medizinische Monatsschrift fur Pharmazeuten*.

[2] Sakazaki F, Kataoka H, Okuno T (2007). Ozonated olive oil enhances the growth of granulation tissue in a mouse model of pressure ulcer. *Ozone: Science and Engineering*.

[3] Matsumoto A, Sakurai S, Shinriki N, Suzuk S, Miura T (2000). Therapeutic effects of ozonized olive oil in the treatment of intractable fistula and wound after surgical operation. *Journal of Japan Surgical Association*.

[4] Kagawa Y (2001). *Standard Tables of Food Composition in Japan*.

[5] Criegee R (1975). Mechanism of ozonolysis. *Angewandte Chemie—International Edition*.

[6] Miura T, Iwai A, Tamoto K, Yamazaki A, Nochi H Components and ant-inflammatory action mechanism of ozonized olive oil.

[7] Schulz S (1981). A new model for integral measuring of wound healing processes in small laboratory animals, tested with ozonized olive oil. *Deutsche Tierarztliche Wochenschrift*.

[8] Sakurai S (2001). Physicochemical property and clinical application of ozonated olive oil. *Pharm Tech Japan*.

[9] (2007). Standard practice for testing biomaterials in rabbits for primary skin irritation. *ASTM F719-81*.

[10] Pharmaceutical Society of Japan (1995). *Standard Methods of Analysis for Hygienic Chemists*.

[11] Vasina V, Abu-Gharbieh E, Barbara G (2008). The *β*3-adrenoceptor agonist SR58611A ameliorates experimental colitis in rats. *Neurogastroenterology and Motility*.

[12] Ohkawa H, Ohishi N, Yagi K (1979). Assay for lipid peroxides in animal tissues by thiobarbituric acid reaction. *Analytical Biochemistry*.

[13] Lowry OH, Rosebrough NJ, Farr AL, Randall RJ (1951). Protein measurement with the Folin phenol reagent. *The Journal of Biological Chemistry*.

[14] Harris ML, Schiller HJ, Reilly PM, Donowitz M, Grisham MB, Bulkley GB (1992). Free radicals and other reactive oxygen metabolites in inflammatory bowel disease: cause, consequence or epiphenomenon?. *Pharmacology & Therapeutics*.

[15] Van der Vliet A, Bast A (1992). Role of reactive oxygen species in intestinal diseases. *Free Radical Biology and Medicine*.

[16] Grisham MB, Granger DN (1988). Neutrophil-mediated mucosal injury: role of reactive oxygen metabolites. *Digestive Diseases and Sciences*.

[17] Cuzzocrea S, Zingarelli B, Hake P, Salzman AL, Szabo C (1998). Antiinflammatory effects of mercaptoethylguanidine, a combined inhibitor of nitric oxide synthase and peroxynitrite scavenger, in carrageenan-induced models of inflammation. *Free Radical Biology and Medicine*.

[18] Keshavarzian A, Morgan G, Sedghi S, Gordon JH, Doria M (1990). Role of reactive oxygen metabolites in experimental colitis. *Gut*.

[19] Seguí J, Gironella M, Sans M (2004). Superoxide dismutase ameliorates TNBS-induced colitis by reducing oxidative stress, adhesion molecule expression, and leukocyte recruitment into the inflamed intestine. *Journal of Leukocyte Biology*.

[20] Hawkins JV, Emmel EL, Feuer JJ (1997). Protease activity in a hapten-induced model of ulcerative colitis in rats. *Digestive Diseases and Sciences*.

[21] Nieto N, Fernandez MI, Torres MI, Ríos A, Suarez MD, Gil A (1998). Dietary monounsaturated n-3 and n-6 long-chain polyunsaturated fatty acids affect cellular antioxidant defense system in rats with experimental ulcerative colitis induced by trinitrobenzene sulfonic acid. *Digestive Diseases and Sciences*.

[22] Halliwell B (1991). Reactive oxygen species in living systems: source, biochemistry, and role in human disease. *The American Journal of Medicine*.

[23] Halliwell B (1992). Reactive oxygen species and the central nervous system. *Journal of Neurochemistry*.

[24] Loguercio C, D'Argenio G, Delle Cave M (1996). Direct evidence of oxidative damage in acute and chronic phases of experimental colitis in rats. *Digestive Diseases and Sciences*.

[25] Zamora Rodríguez ZB, González Álvarez R, Guanche D (2007). Antioxidant mechanism is involved in the gastroprotective effects of ozonized sunflower oil in ethanol-induced ulcers in rats. *Mediators of Inflammation*.

[26] Bailey PS (1978). *Ozonation in Organic Chemistry*.

[27] Díaz M, Lezcano I, Molerio J, Hernández F (2001). Spectroscopic characterization of ozonides with biological activity. *Ozone: Science & Engineering*.

[28] Geweely NSI (2006). Antifungal activity of ozonized olive oil (Oleozone). *International Journal of Agriculture and Biology*.

[29] Bilici D, Süleyman H, Banoglu ZN (2002). Melatonin prevents ethanol-induced gastric mucosal damage possibly due to its antioxidant effect. *Digestive Diseases and Sciences*.

[30] Kanter M, Demir H, Karakaya C, Ozbek H (2005). Gastroprotective activity of Nigella sativa L oil and its constituent, thymoquinone against acute alcohol-induced gastric mucosal injury in rats. *World Journal of Gastroenterology*.

